# BMP Signaling in Astrocytes Downregulates EGFR to Modulate Survival and Maturation

**DOI:** 10.1371/journal.pone.0110668

**Published:** 2014-10-17

**Authors:** Anja R. Scholze, Lynette C. Foo, Sara Mulinyawe, Ben A. Barres

**Affiliations:** 1 Stanford University School of Medicine, Department of Neurobiology, Stanford, California, United States of America; 2 Institute of Molecular and Cell Biology, A *Star, Singapore, Singapore; Laboratoire de Biologie du Développement de Villefranche-sur-Mer, France

## Abstract

Astrocytes constitute a major cell population in the brain with a myriad of essential functions, yet we know remarkably little about the signaling pathways and mechanisms that direct astrocyte maturation. To explore the signals regulating astrocyte development, we prospectively purified and cultured immature postnatal rodent astrocytes. We identified fibroblast growth factors (FGFs) and bone morphogenetic proteins (BMPs) as robust trophic factors for immature astrocytes. We showed that astrocytes respond directly to BMPs via phosphorylation of the smad1/5/8 pathway. *In vitro*, BMP signaling promoted immature astrocytes to adopt multiple characteristics of mature astrocytes, including a more process-bearing morphology, aquaporin-4 (AQP4) and S100β immunoreactivity, limited proliferation, and strong downregulation of epidermal growth factor receptor (EGFR). *In vivo,* activation of the smad1/5/8 pathway in astrocytes was seen during early postnatal development, but inhibition of astrocyte proliferation was not observed. These insights can aid in the further dissection of the mechanisms and pathways controlling astrocyte biology and development.

## Introduction

Astrocytes constitute a major cell population in the central nervous system (CNS). They have been shown to modulate a myriad of essential CNS processes including brain homeostasis, blood flow regulation, neurotransmission, synaptic development and injury response [1], [2]. Despite their central importance, astrocyte specification, maturation, and heterogeneity are still not well understood.

In rodent brains, astrogenesis begins around birth and is largely complete by the end of the second postnatal week [3]. Early astrogenesis in cortical development is largely driven by the specification of radial glia and subventricular zone (SVZ) progenitors, but as the cortex grows, local astrocyte division becomes another major source [4], [5]. The final number, complexity and spatial organization of astrocytes are tightly controlled to achieve a properly functioning brain [6], yet we know remarkably little about signaling pathways and mechanisms that direct immature astrocytes to become mature and stop dividing.

Both extrinsic and intrinsic factors contribute to astrogenesis [7]. Like neurons and other glia, developing astrocytes are dependent on external survival signals or they die by apoptosis [8]–[10]. In the developing postnatal brain, peak astrocyte apoptosis occurs concurrent with maximal astrocyte generation, suggesting that one possible mechanism regulating the final cell number and localization could be a limiting survival cue.

Currently, known trophic factors for immature astrocytes also act as mitogens [10]. Appropriately, early in development newly formed astrocytes divide readily until they approach a plateau number between postnatal days 7 and 10. By postnatal day 14, however, astrocyte proliferation *in* *vivo* is essentially complete and mature astrocytes exhibit very limited division in the healthy CNS [3], [11]. This fact makes it unlikely that mature astrocyte survival remains linked to proliferation and suggests that yet unknown signals can restrict astrocyte proliferation. Alternatively, astrocytes could modulate their intrinsic response to, or reliance on, trophic and mitotic factors as they mature.

Until recently, dissection of the signals governing astrocyte development has been complicated by the absence of a method to culture astrocytes in a non-reactive state reflective of their normal *in* *vivo* properties. Here we employ a recently developed protocol for the prospective purification and defined culture of postnatal rodent astrocytes [10] to identify novel survival cues, including bone morphogenetic proteins (BMPs) and fibroblast growth factors (FGFs). BMP signaling had robust and reliable trophic effects on immature astrocytes. We showed that immature astrocytes respond directly to BMPs via phosphorylation of the smad1/5/8 pathway, and activation of this pathway was seen *in* *vivo* during early postnatal development. *In vitro*, BMP signaling modulated astrocyte morphology, maturation, proliferation and expression of epidermal growth factor receptor (EGFR). These insights can help us further understand the complex mechanisms and pathways controlling astrocyte development.

## Results

### Identification of BMPs and FGFs as trophic factors for purified astrocytes

Purified immature astrocytes grown in serum-free media are dependent on exogenous trophic factors or they die by apoptosis. Several growth factors, including heparin-binding epidermal growth factor (HbEGF), transforming growth factor alpha (TGFα), and Wnt7a have been shown to promote astrocyte survival *in* *vitro* [10]. However, that list is not exhaustive, as these signals are insufficient to keep 100% of astrocytes alive. Furthermore, isolated vascular cells can promote astrocyte survival, at least partially, by an unique signal [10].

**Figure 1:**
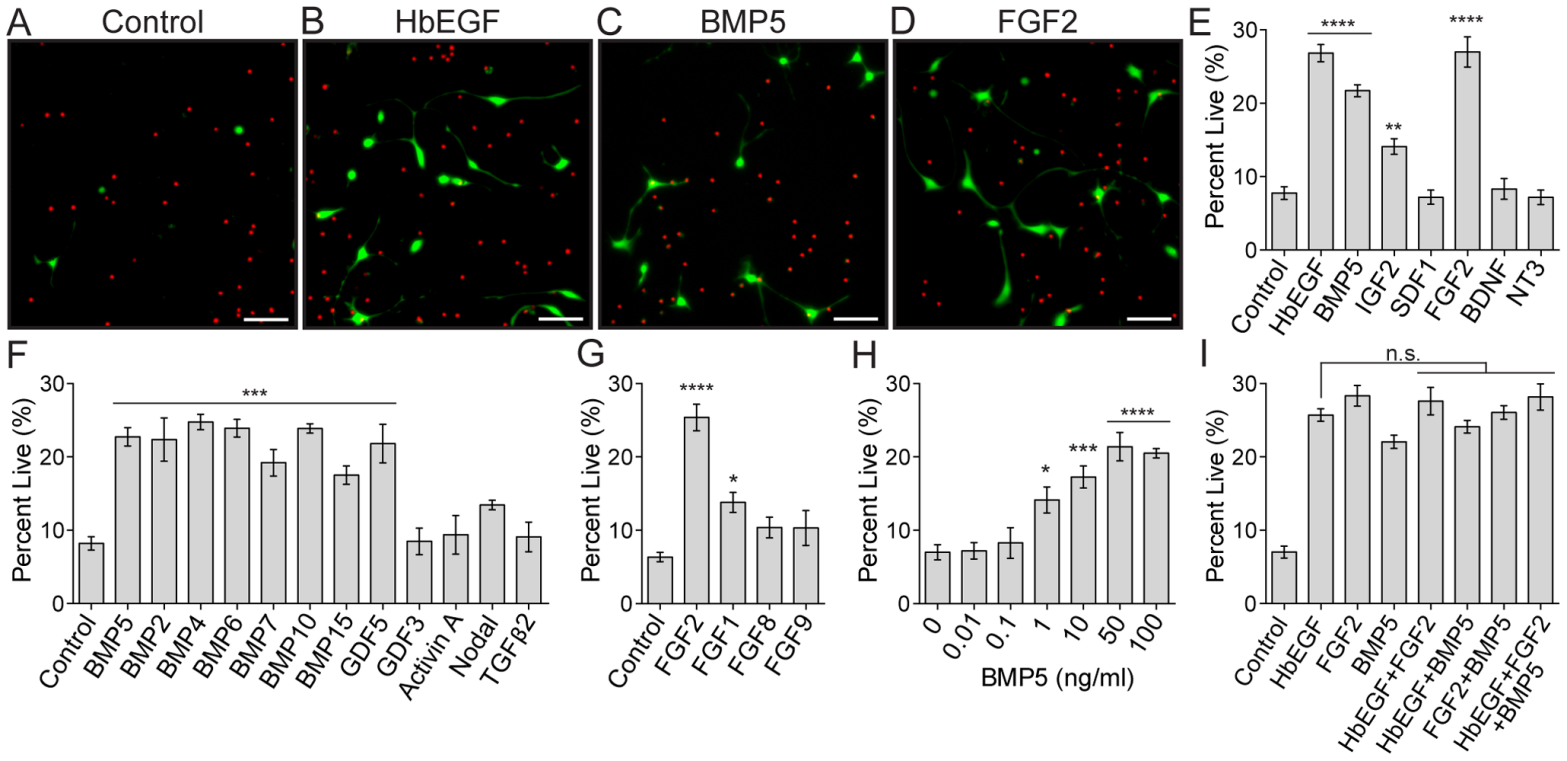
BMP and FGF are robust trophic factors for purified astrocytes. A–D. Live/Dead viability images at 3DIV of purified astrocyte cultures with control base media (A) containing either HbEGF (B), BMP5 (C), or FGF2 (D). Green cells are alive, red cells are dead. Scale bars are 100 µm. E–I. Quantification of purified astrocyte survival at 3DIV in base media with the addition of various cytokines. 5 ng/ml HbEGF (positive control), as well as 100 ng/ml BMP5, 100 ng/ml IGF2 and 10 ng/ml FGF2 each significantly increase astrocyte viability above control (E). Within the TGF-β superfamily multiple BMP subfamily members also promoted astrocyte survival at 100 ng/ml (F). Additional FGF family members were examined, but only FGF1 also had a modest trophic effect at 10 ng/ml (G). BMP5 survival was dose dependent and trophic effects plateau at 50 ng/ml (H). No additive effects on astrocyte survival above HbEGF alone were observed with any combination of HbEGF, FGF2, and BMP5 (I). N ≥3 for each condition. Significance determined using one-way ANOVA with Dunnett correction. Error bars represent SEM.

To identify additional trophic factors, postnatal astrocytes were isolated from P7 rat cortices by immunopanning and cultured in a defined media (see Experimental Procedures). Astrocytes were plated at a low density to prevent accumulation of autocrine and paracrine survival signals, such as astrocyte-derived HbEGF. Under these base conditions, only 7.8% ±0.9% of cells remained alive after 3 days *in* *vitro* (DIV), as measured by Calcein AM staining (Fig. 1A and E). The ability of various cytokines to enhance astrocyte survival was assayed in media containing 0.5 µg/ml of aphidicolin [10], an inhibitor of cell division, to eliminate any confounding mitotic effects.

Candidate survival factors were selected using cell-type specific gene expression data sets previously generated in our lab [12], [13]. A list of receptors highly expressed by astrocytes was initially used to suggest numerous ligands. To identify potential vascular cues, we refined the list by enriching for cytokines strongly expressed in pericytes, including BMP5, insulin-like growth factor II (IGF2), and stromal cell-derived factor 1 (SDF1/CXCL12) [14]. In an initial screen, a significant enhancement of astrocyte survival over control base media was observed with both 10 ng/ml of fibroblast growth factor 2 (FGF2) (27.0±2.1% alive, p<0.0001) and 100 ng/ml of BMP5 (21.7±0.8% alive, p<0.0001) (Fig. 1C–E). These survival effects were similar in magnitude to the known astrocyte trophic factor HbEGF (26.8±1.2% alive). 100 ng/ml IGF2 also exhibited a modest, but significant, effect on astrocyte survival (14.1±1.1% alive, p<0.01). SDF1, another vascular secreted factor, did not significantly promote astrocyte survival. Brain-derived neurotrophic factor (BDNF) and neurotrophin-3 (NT-3) also failed to keep astrocytes alive, despite high astrocyte expression of their cognate receptors (Fig. 1E).

The FGF family of growth factors is multi-membered [15] and astrocytes express several FGF receptors, including very high levels of FGFR3 [12]. Therefore, we tested the survival-promoting ability of other FGF family members (Fig. 1G). Culture in 10 ng/ml of FGF1 resulted in a significant increase in astrocyte survival, but this effect was very modest (13.8±1.4% alive, p<0.05). Neither 10 ng/ml of FGF8 or FGF9 was sufficient to significantly promote astrocyte survival at 3DIV. Surprisingly, within the FGF family a robust trophic effect on purified astrocytes is largely limited to FGF2.

The transforming growth factor (TGF)-beta superfamily, of which BMP5 is a member, is also very expansive [16]. When assayed in our low-density cultures, numerous other TGF-β superfamily members were also sufficient to promote astrocyte survival at 100 ng/ml (Fig. 1F). This included BMP2 (22.4±3.0% alive), BMP4 (24.8±1.0% alive), BMP6 (23.9±1.2% alive), BMP7 (19.2±1.8% alive), BMP10 (23.9±0.6% alive), BMP15 (17.5±1.2% alive) and growth differentiation factor 5 (GDF5) (21.8±2.6% alive)**.** Each of these factors was similarly effective at promoting astrocyte survival as compared to BMP5 (p>0.05). Another subset of TGF-β superfamily members, however, did not significantly improve astrocyte survival. These included GDF3, Activin A, Nodal and TGFβ2. TGF-β superfamily enhancement of astrocyte survival is largely restricted to the BMP subfamily, within which multiple members are effective.

The response to BMP5 was dose dependent and saturable. A significant increase in astrocyte survival in response to BMP5 was detected at concentrations as low as 1 ng/ml (14.1±1.8% alive, p<0.05) and maximal survival effects plateaued at 50 ng/ml (21.4±1.9% alive, p<0.0001) (Fig. 1H). No additive effects on astrocyte survival above HbEGF alone were observed with any combination of HbEGF, FGF2, and BMP5 (Fig. 1I). This result suggests that important survival cues are still missing from our culture system that could be acting through a unique mechanism or on a subpopulation of the immature astrocytes.

BMP5 and BMP6 are both highly expressed by CNS pericytes [13], [14], and almost all astrocytes have endfeet that contact the vasculature by the end of the second postnatal week [10]. This suggests that BMPs are temporally and spatially relevant during immature astrocyte development *in* *vivo*. We chose to further investigate the role of BMP signaling in astrocyte maturation using BMP5, as it was a robust and reliable astrocyte survival factor.

### Astrocytes signal intracellularly via phosphorylation of smads1/5/8 in response to BMPs

Previous literature has shown that BMPs play a role in the specification of astrocytes from neural progenitor cells (NPCs) *in* *vitro* and *in* *vivo* [17]–[20]; however, the direct effects of BMP on immature and mature astrocytes are unknown. BMPs signal by binding to heterotetrameric serine/threonine kinase receptor complexes, which are comprised of type I and type II receptors. Activated type I receptors phosphorylate smad family transcription factors. There are 8 smad proteins which mediate signaling downstream of TGFβ-superfamily ligands. Smads 1, 5 and 8 signal concurrently, typically downstream of BMPs, while smads 2 and 3 mediate TGFβ responses. Phosphorylated smads from either group (1/5/8 or 2/3) associate with a common mediator, smad4, to transactivate target genes [16].

**Figure 2:**
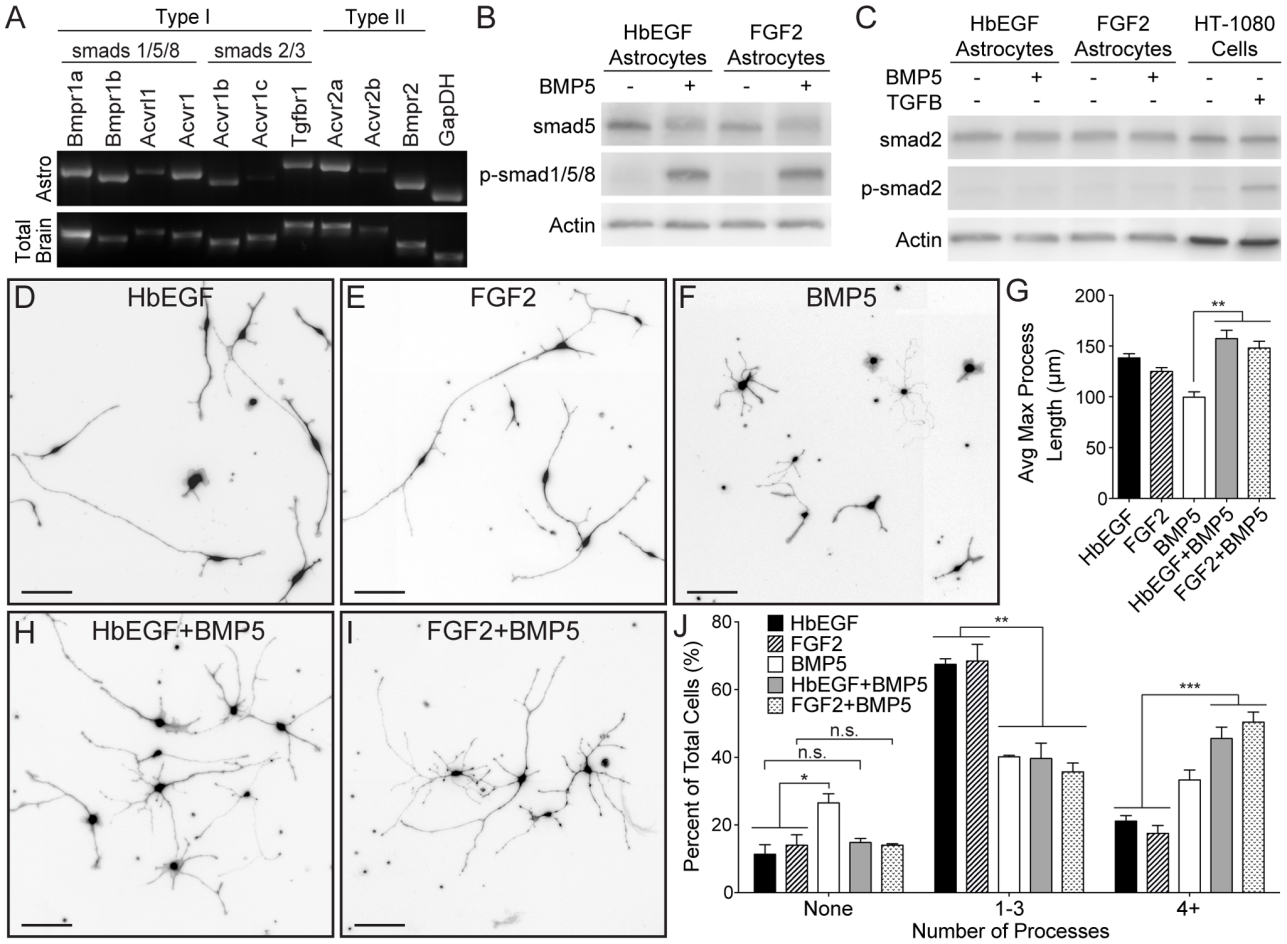
BMP signaling activates the smad1/5/8 pathway in astrocytes and promotes a more process-bearing morphology. A. Semi-quantitative RT-PCR of acutely purified P7 rat astrocytes confirmed *in* *vivo* expression of numerous TGF-β superfamily Type I and Typ1 II receptors. B–C. Western blots of purified astrocytes cultured in either HbEGF or FGF2 and then treated with 50 ng/ml of BMP5 for 2 hours show robust activation of the smad1/5/8 pathway (p-smad1/5/8) (B), but not the smad2/3 pathway (p-smad2) (C). TGF-β treated HT-1080 Cell Extract was used as a positive control for detection of smad2/3 pathway activation. Actin bands confirm equal protein loading. D–F and H–I. Images of purified astrocyte morphology using CellMask HCS staining to visualize cell membranes. Astrocytes were cultured for 3DIV in HbEGF (D), FGF2 (E), BMP5 (F), HbEGF+BMP5 (H), or FGF2+BMP5 (I). G and J. Quantification of astrocyte morphology was done using Sholl Analysis in ImageJ. Average maximum process length (G) and number of primary processes per cell (J) were analyzed. N = 3 for each condition. Significance determined using one-way ANOVA with Tukey correction. Error bars represent SEM.

At the peak of postnatal generation astrocytes expressed multiple of TGFβ-superfamily receptors, as shown by semi-quantitative RT-PCR of acutely isolated rat astrocytes (Fig. 2A). At the mRNA level, highly expressed type I receptors included Bmpr1a, Bmpr1B and Acvr1, all of which typically signal through the phosphorylation of smads 1/5/8. For type II receptors, Acvr2a and Bmpr2 mRNAs were highly expressed. These results confirmed what has been seen by gene chip analysis [12]. Postnatal immature astrocytes are therefore capable of responding to various TGFβ-superfamily ligands, but smad1/5/8 pathway receptors are most highly represented.

To identify which smads signal downstream of BMP in immature astrocytes, purified astrocytes were treated for 2 hours with either BMP or a 4 mM HCl vehicle control. Cell lysates were examined by immunoblotting for smad phosphorylation. Independent of whether the astrocytes were kept alive with HbEGF or FGF2 prior to treatment, 50 ng/ml of BMP5 strongly stimulated phosphorylation of the smad1/5/8 pathway (Fig. 2B). Smad 2, however, remained unphosphorylated (Fig. 2C). BMP4 and 6 also stimulated smad1/5/8 phosphorylation (Fig. S1A). Altogether, this data demonstrates that immature astrocytes signal intracellularly via the smad1/5/8 pathway in response to BMPs.

### BMPs promote immature astrocytes to become more process bearing

To further understand the effects of BMP signaling on immature astrocytes, we examined cell morphology at 3DIV. Purified astrocytes were cultured at low density in 0.5 µg/ml of aphidicolin to isolate morphology effects. Astrocytes grown in HbEGF or FGF2 exhibited very similar morphologies. Qualitatively, these cells were largely bipolar with elongated cell bodies and long, thick processes (Fig. 2D and E). Conversely, astrocytes cultured in BMP5 were typically more radial with small, rounded cell bodies and shorter, very fine and branched processes (Fig. 2F). A similar effect on morphology has been seen in cultured embryonic SVZ progenitor cells that differentiate into astrocyte lineage cells in response to BMP4 [21]. In our purified postnatal cultures, BMP4, 6 and 10 also all showed similar effects on astrocyte morphology, suggesting that this effect is not specific to BMP5 (Fig. S1B–D).

Cell processes were quantified using Sholl analysis in ImageJ [22]. At 3 DIV, the majority of astrocytes cultured in HbEGF and FGF2 had 1–3 primary processes (67.5.1±1.6% and 68.5±4.9%, respectively), while relatively few had 4 or more. In comparison, when cultured in BMP5, the percentage of astrocytes with 1–3 primary process decreased significantly to 40.1±0.5% (p<0.01) and the percentage with 4 or more increased (33.3±2.9%) (Fig. 2J). The concurrent addition of BMP5 to cultures containing HbEGF or FGF2 generated astrocytes with morphological qualities reminiscent of those seen in BMP5 alone conditions (Fig. 2H and I). Using these concurrent cultures, we quantified the combinatorial effects of HbEGF+BMP5 and FGF2+BMP5 on astrocyte morphology. The fraction of astrocytes with four or more processes was significantly higher when grown in HbEGF+BMP5 (45.5±3.4%, p<0.001) or FGF2+BMP5 (50.3±3.0%, p<0.001) as compared to HbEGF or FGF2 alone (21.1±1.6% and 17.5±2.3%, respectively) (Fig. 2J). Thus, BMP signaling in immature astrocytes promotes a more process-bearing morphology at 3 DIV, and this effect can act dominantly in the presence of other trophic signals. Since mature astrocytes *in* *vivo* are highly process bearing cells, these results suggest that BMP signaling could be promoting or accelerating the maturation of immature astrocytes.

Although BMP5 is sufficient to keep astrocytes alive, cell morphology in these cultures was more variable and less thriving than in cultures containing only HbEGF or FGF2. This was illustrated by quantification of primary process number and average maximum process length. Concurrent with the previously described decrease in fraction of astrocytes with 1–3 process and increase in fraction of astrocytes with 4 or more, culture in BMP5 also increased the number of astrocytes with no processes (26.5±2.7%) (Fig. 2J). These non-process bearing astrocytes appeared healthy by calceinAM staining (Fig. 1C), but displayed a flat, amoeboid shape (Fig. 2F). Thus, culture of astrocytes in BMP5 alone broadens the range of primary process number observed. Concurrent culture in either HbEGF+BMP5 or FGF2+BMP5 negated the increase in non-process bearing astrocytes seen with BMP5 alone while further increasing the fraction with 4 or more (Fig. 2J). Additionally, astrocytes cultured in BMP5 had a significantly shorter average process length (99.7±5.3 µm, p<0.01) than astrocytes cultured in HbEGF (138.3±4.1 µm). Combinatorial culture in HbEGF+BMP5 (157.4±8.2 µm) or FGF2+BMP5 (148.2±6.25 µm) significantly increased process length as compared to BMP5 alone (p<0.001) (Fig. 2G). In fact, astrocyte process length in these combinatorial cultures was no longer significantly different from HbEGF alone. Astrocytes cultured in BMP5 plus either HbEGF or FGF2 exhibit a more uniform increase in primary process number and length than BMP5 alone. Together, these data suggest that, although BMP signaling is sufficient to keep astrocytes alive, combining BMP5 with another growth factor (HbEGF or FGF2) makes the effects on morphology more robust and penetrant.

### BMP signaling promotes maturation and upregulates GFAP expression in immature astrocytes *in* *vitro*

**Figure 3:**
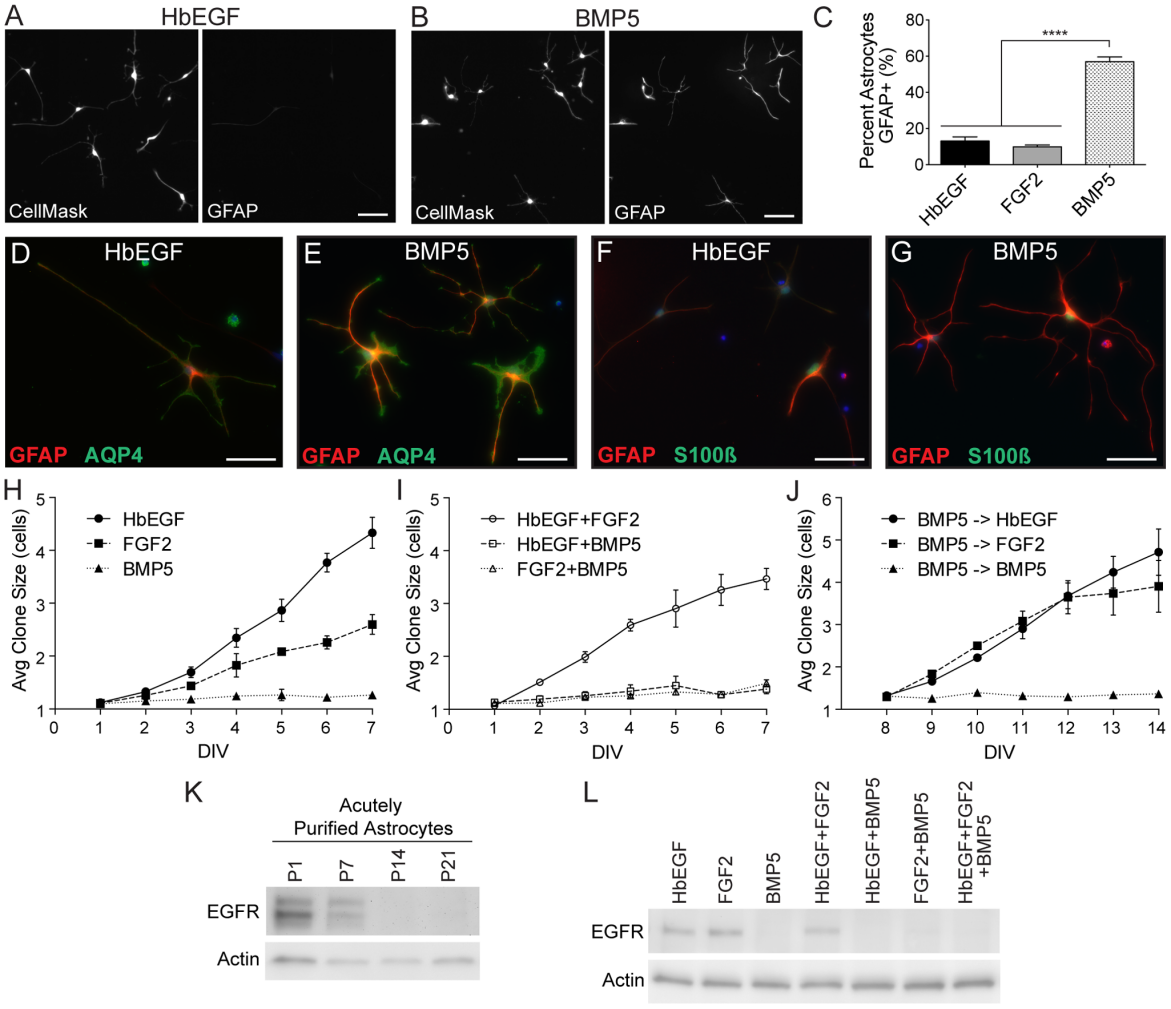
BMP signaling modulates astrocyte maturation *in* *vitro*. A–C. Purified astrocytes were cultured for 3DIV in HbEGF (A), BMP5 (B), or FGF2 and GFAP expression examined by immunostaining. CellMask HCS staining was used to visualize all astrocytes. Images show individual channels from the same field. Scale bars are 100 µm. The percentage of astrocytes expressing GFAP at 3DIV was quantified using ImageJ (C). D–G. Purified astrocytes were cultured for 3DIV in HbEGF or BMP5 and the expression of AQP4 and S100ß examined by immunostaining. All cultures were also stained with GFAP. Scale bars are 50 µm. H–J. The proliferative capabilities of purified astrocytes in HbEGF, BMP5 and FGF2 were quantified *in* *vitro* over 7 days using clonal analysis. Average clone size was calculated for both individual growth factors (H) and combinations of factors (I). Clonal analysis was also performed on astrocytes pre-treated with BMP5 for 7DIV to examine reversibility (J). K–L. Western blots for EGFR. Protein samples collected from acutely purified astrocytes at different postnatal time points confirm strong developmental downregulation of astrocyte EGFR (K). EGFR is also strongly downregulated in astrocytes cultured with BMP5 for 6DIV, even in the presence of other growth factors (L). Actin bands confirm equal protein loading. N ≥3 for each condition. Significance determined using one-way ANOVA with Tukey correction. Error bars represent SEM.

The intermediate filament glial fibrillary acidic protein (GFAP) is a classic astrocyte marker that is expressed at different levels by mature astrocytes in different brain regions [23]. Astrocyte GFAP expression also varies across development and after injury [24]. Previous work has identified a link between BMP stimulation of NPCs and increased GFAP expression in the derived astrocyte lineage cells [19], [25], [26]. To quantify the direct effects of BMP signaling on GFAP expression in immature astrocytes, we employed immunostaining. Culture in BMP5 significantly increased the intensity of GFAP staining in astrocytes, as well as the fraction that expressed GFAP at 3DIV (56.9±2.6%, p<0.0001) as compared to either HbEGF (13.1±2.3%) or FGF2 (9.9±1.0%) (Fig. 3A–C). Thus, BMP signaling can directly increase GFAP expression in immature astrocytes, although our understanding of what variation in GFAP expression means for astrocyte maturation and function is still fairly limited.

To further characterize our cultures, we immunostained with additional astrocyte markers as well as markers for other brain cell types. At 3DIV, astrocytes cultured in BMP5 showed strong immunoreactivity for the astrocyte markers AQP4 and S100β (Fig. 3D–G), but did not display staining for markers of other differentiated cell types including neurons and oligodendrocyte-lineage cells (Fig. S2E–L). Remarkably, in BMP5 cultures, AQP4 staining was often found to be highly concentrated at the endfeet of astrocytes, suggesting that they may develop some features of normal polarization despite the absence of vascular cells. Markers to definitively differentiate between NPCs and proliferating astrocytes are currently limited, but we did examine the classic neural progenitor markers Sox2 and Nestin (Fig. S2A–D). It is important to note, however, that these do not exclusively mark precursor cells, as expression of both has been previously reported for astrocytes *in* *vivo* [27]–[29]. Along those lines, we observed weak staining for Sox2 in astrocytes cultured in either HbEGF or BMP5. This is consistent with RNA-seq data generated in our lab, which reports fairly high levels of Sox2 mRNA in postnatal astrocytes [14]. Astrocytes cultured in HbEGF also displayed Nestin immunoreactivity, whereas this staining was almost completely absent in BMP5-treated astrocytes. Together, this data confirms that BMP signaling does not convert purified astrocytes to another cell type and suggests that it promotes several aspects of astrocyte maturation.

### Astrocyte proliferation is actively and reversibly inhibited by BMP signaling *in* *vitro*

Purified astrocytes cultured in HbEGF divide relatively slowly at a rate of approximately once every 3 days [10]. In the healthy CNS, however, mature astrocytes exhibit even lower rates of division [3], [11]. The radial, process-bearing morphology of astrocytes cultured in BMP5 suggested that these cells might be less mitotically active than their bipolar HbEGF and FGF2 counterparts. Additionally, previous work has demonstrated that BMP stimulation of neural stem cells can cause them to reversibly exit from the cell cycle [30], [31]. To understand if BMP signaling modulates this aspect of astrocyte maturation we examined proliferation *in* *vitro*.

To directly assess proliferative capacity, we plated acutely purified astrocytes at extremely low density to achieve spatially isolated single cell clones. The number of cells per clone was counted every 24 hours for 7DIV. As expected, the clone size of astrocytes in HbEGF increased gradually over the 7 day period to achieve a final average size of 4.3±0.3 cells. In presence of FGF there was a more modest increase in average clone size to 2.6±0.2 cells at 7DIV. This data indicates that FGF2 promotes astrocyte division at approximately half the rate of HbEGF, or about one division every 5 days. Immature astrocytes cultured in BMP5 almost never divided, and the average clone size at 7DIV (1.3±0.6 cells) was not significantly different than at 1DIV (1.1±0.2 cells) (Fig. 3H). Equivalent results were also obtained with BMP4 and BMP6, so this effect is not specific to BMP5 (Fig. S1E).

There are two possible explanations for the lack of astrocyte mitosis in BMP5 cultures: BMPs could not be mitogenic, or BMP signaling could be actively inhibiting astrocyte proliferation. To differentiate between these possibilities, clonal analysis was performed with combinations of growth factors to see which proliferative effect was dominant. Intriguingly, the simultaneous addition of BMP5 with either HbEGF or FGF2 completely halted astrocyte proliferation. After 7DIV, the average clone size was 1.4±0.1 cells for astrocytes in HbEGF+BMP5 and 1.5±0.1 cells for astrocytes in FGF2+BMP5 (Fig. 3I). These final average clone sizes were not significantly different from their respective 1DIV averages or astrocytes in BMP5 only at 7DIV. Thus, BMP5 exhibits a dominant inhibitory effect on astrocyte proliferation that acts independently of the coincident mitogenic cue. This data demonstrates that BMP signaling actively and effectively inhibits astrocyte proliferation *in* *vitro*.

Next, we examined if BMP5 inhibition of astrocyte proliferation is reversible. Purified astrocytes were cultured for 7DIV in BMP5 to generate quiescent astrocytes. Then, at 7DIV, these cells were trypsinized, replated at clonal density, and clone size recorded for the subsequent week. Even after prolonged culture in BMP5, astrocytes that were moved to either FGF2 or HbEGF containing media immediately resumed proliferating. The average clone size in these cultures gradually increased over the entire 7-day period that astrocytes were exposed to either mitogen, with final 14DIV averages that were significantly different from their initial 8DIV averages (Fig. 3J). The average clone size at 14DIV was 4.7±0.5 cells for astrocytes moved from BMP5 to HbEGF media and 3.9±0.6 cells for astrocytes moved from BMP5 to FGF2 media. The replating process itself did not induce proliferation, as astrocytes moved from BMP5 to BMP5 media remained non-mitotic throughout the 7DIV period. Therefore, BMP inhibition of astrocyte proliferation is readily reversible.

### BMP signaling directly downregulates astrocyte EGFR expression *in* *vitro*

EGFR is an important transducer of signals for many stages of astrocyte development including specification, survival and proliferation [32]–[34]. It is a member of the ErbB family of tyrosine kinase receptors and is activated by ligand-dependent receptor dimerization [35]. Early in development, almost all astrocytes express EGFR, but as development progresses, astrocytes dramatically downregulate it [10], [12], [36]. We asked if BMP signaling could control this change in receptor expression by immature astrocytes.

First, we confirmed EGFR downregulation in cortical rat astrocytes by immunoblotting of acutely purified astrocyte samples isolated at different developmental time points. EGFR was present at high levels in astrocytes isolated at P1 and P7, but was not detected in P14 or P21 astrocytes. Therefore, similar to what has been reported in mice, EGFR is effectively downregulated between P7 and P14 in rat cortical astrocytes (Fig. 3K). To determine the direct effects of BMP signaling on EGFR expression in immature astrocyte, we performed immunoblotting of purified astrocytes cultured for 6DIV with various growth factors. Surprisingly, exposure of immature astrocytes to BMP5, both in isolation and in the presence of HbEGF and/or FGF, almost completely eliminated EGFR expression in all cases (Fig. 3L). This demonstrates that BMP signaling directly and robustly downregulates EGFR in immature astrocytes.

All together, our *in* *vitro* data shows that BMP signaling promotes immature astrocytes to adopt many characteristics typical of more mature astrocytes, such as cessation of proliferation with continued viability, upregulation of astrocyte markers, elaboration of fine processes, and downregulation of EGFR. Therefore, we hypothesized that BMP signaling during astrocyte development *in* *vivo* could be a way to coordinate these multiple aspects of astrocyte maturation that, until now, have seemed irreconcilable.

### Cortical astrocytes display smad1/5/8 pathway activity during early postnatal development *in* *vivo*

**Figure 4:**
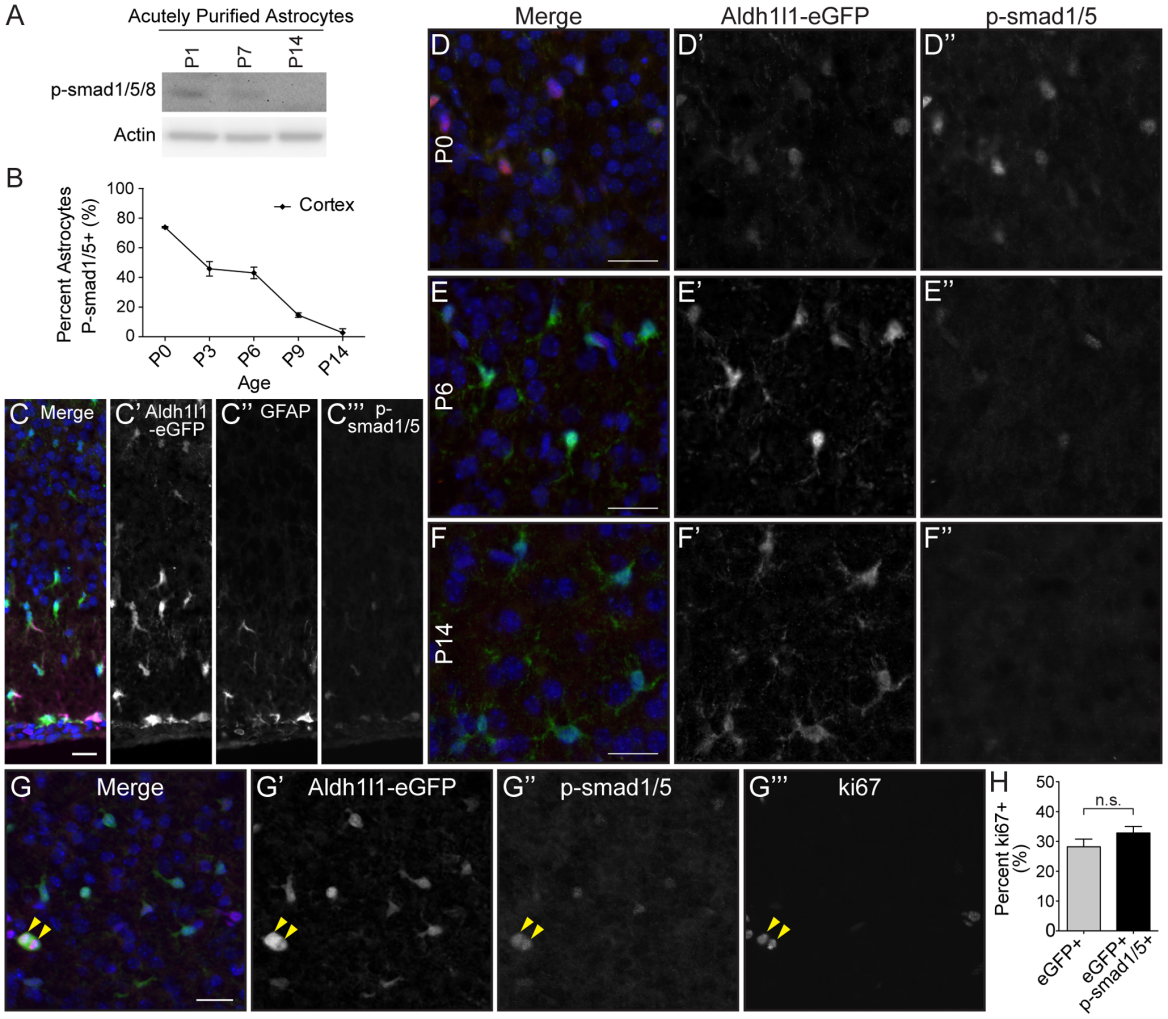
Cortical astrocytes *in* *vivo* display smad1/5/8 pathway activation during early postnatal development. A. Western blotting for p-smad1/5/8 in acutely purified astrocytes across developmental time points demonstrates pathway activation during the early postnatal period. Actin bands confirm equal protein loading. B–F. Immunostaining of sagittal brain sections from Aldh1l1-eGFP mice to visualize astrocytes. Merged images show DAPI (blue), p-smad1/5 (red) and Aldh1l1-eGFP (green) (D–F). Colocalization of p-smad1/5 with eGFP+ cortical astrocytes was quantified across the first two weeks of postnatal development using ImageJ (B). N = 2 animals for each time point. Immunostaining of P6 brains sections with DAPI (blue), p-smad1/5 (red), GFAP (magenta) and Aldh1l1-eGFP (green) shows that p-smad1/5 and GFAP staining localizes predominately to astrocytes in the outer cortical layers and glia limitans (C). G–H. Sagittal brain sections from Aldh1l1-eGFP mice were sequentially immunostained for p-smad1/5 and ki67. Yellow arrows indicate cortical astrocytes that express both p-smad1/5 and ki67 (G). Colocalization of p-smad1/5 and ki67 with eGFP+ cortical astrocytes was quantified using ImageJ (H). N = 3 animals. Significance determined using students t-test. All scale bars are 25 µm. Error bars represent SEM.

To examine the role of BMP signaling in immature astrocyte development *in* *vivo*, we used smad1/5/8 pathway phosphorylation to identify BMP-responsive cells. Acutely purified cortical rat astrocytes from different postnatal developmental time points were analyzed by immunoblotting. Phosphorylated-smad1/5/8 (p-smad1/5/8) was detected at low levels in acutely purified P1 and P7 astrocytes, but was absent in P14 astrocytes (Fig. 4A). Thus, the smad1/5/8 pathway is active in cortical astrocytes during the first postnatal week of development, suggesting that they may be responding to an extrinsic BMP signal.

To confirm that the isolation process was not impacting phospho-protein levels in astrocytes, smad1/5/8 pathway activation was also examined in the developing postnatal cortex by immunostaining. Cryosections were generated from Aldh1l1-eGFP mice, in which most or all astrocytes express eGFP [12]. Phosphorylated-smad1/5 (p-smad1/5) staining was used to localize smad1/5/8 pathway activation. P-smad1/5 was detected in the nuclei of a subset of eGFP+ cortical astrocytes during early postnatal development. Maximal colocalization between astrocytes and p-smad1/5 was observed at postnatal day 0 (73.9% ±0.5%) (Fig. 4D). The percentage of p-smad1/5+ astrocytes decreased steadily at progressively later developmental time points (Fig. 4B). At P6, the percentage had declined to 43.1±3.9% of astrocytes and by P14 it was 2.7±2.7% (Fig. 4E and F). The immunostaining results corroborate the time course of smad1/5/8 pathway activation in cortical astrocytes seen by immunoblotting.

The strongest p-smad1/5 staining was consistently observed in the outer layers of the cortex, in particular in layer 1 astrocytes. At P0, when the majority of cortical astrocytes expressed p-smad1/5, this pattern was less striking due to the relatively strong expression throughout all cortical layers. The outside-in cortical expression pattern persisted across early postnatal development, becoming more striking as the percentage of p-smad1/5+ astrocytes dwindled (Fig. 4C). By P9, eGFP+ p-smad1/5+ cortical astrocytes were almost exclusively in layer 1. Interestingly, GFAP immunostaining in the developing cortex displayed a very similar localization. GFAP expression in cortical astrocytes appeared first and remained highest in layer 1 astrocytes and the glia limitans (Fig. 4C). Thus, there is an overall positive correlation between the intensity and duration of p-smad1/5 staining in developing cortical astrocytes and GFAP expression.

### P-smad1/5 positive astrocytes *in* *vivo* exhibit proliferative capabilities

A large number of cortical astrocytes are generated during postnatal development by local astrocyte proliferation [5]. To examine the *in* *vivo* relationship between immature astrocyte proliferation and smad1/5/8 pathway activation, we performed immunostaining on brain sections from P3 Aldh1l1-eGFP mice. This time point has both substantial astrocyte proliferation [3] and astrocyte p-smad1/5 expression. Ki67 staining was used to mark mitotically active cells. In the cortex at P3, 27.9±2.8% of all eGFP+ astrocytes and 33.2±2.4% of eGFP+psmad1/5+ astrocytes costained with ki67 (Fig. 4G and H). These data indicate that proliferation is not significantly altered in cortical astrocytes with active smad1/5/8 signaling at P3. This result is perhaps not surprising considering the majority of cortical astrocytes are p-smad1/5+ at P0, yet astrocytes are known to be very actively dividing during the first week of postnatal development.

All together, these results suggest that, *in* *vivo*, immature cortical astrocytes activate the smad1/5/8 pathway in response to a BMP signal that is strongest during early postnatal development. However, P3 astrocytes with ongoing smad1/5/8 pathway activation do not exhibit inhibited proliferation as is observed *in* *vitro* in response to BMP signaling. This suggests that perhaps other cues are present *in* *vivo* that can modulate astrocyte interpretation of BMP signaling or dominantly modulate proliferation. Characterization of this BMP signal, its source, and integrated impacts on astrocyte maturation *in* *vivo* will be interesting areas for further study.

## Discussion

We have shown a robust effect of BMPs on immature astrocytes *in* *vitro* through phosphorylation of the smad1/5/8 pathway. BMPs acted as trophic factors and also promoted astrocytes to adopt multiple characteristics of mature astrocytes including a process-bearing morphology, increased expression of astrocyte markers, limited proliferation, and strong downregulation of EGFR. *In vivo*, immature cortical astrocytes also displayed active smad1/5/8 signaling, although, this was predominately seen very early in postnatal development.

### BMP signaling in astrocyte induction and maturation

During postnatal cortical development, the majority of immature astrocytes show smad1/5/8 pathway activation at birth. BMPs are known to play a role in specification of astrocytes from NPCs during late embryonic development [17], [19], [20], [37], [38], which raises the possibility that neonatal smad1/5/8 activation could be residual phosphorylation from specification signaling. Smad1/5/8 pathway activation, however, is also maintained in close to 40% of cortical astrocytes, mostly in the outer cortical layers, throughout the first postnatal week, which suggests a unique role in immature astrocytes.

BMP signaling in immature cortical astrocytes seems to be interpreted or modulated differently in the context of the CNS environment. Smad1/5/8 pathway activity in astrocytes steadily declines across postnatal development until it is effectively absent at P14. This demonstrates that immature astrocytes *in* *vivo* are BMP responsive, but peak signaling through the smad pathway precedes the normal time course of many maturation events that BMPs were capable of regulating *in* *vitro*, including EGFR downregulation and attenuation of proliferation. There are multiple known non-canonical pathways, however, that can also signal downstream of BMP receptors. These include several MAK kinase pathways as well as PI3K signaling [39]–[42]. It has also been shown that BMPRII can signal directly to Rho-GTPases to cause changes in the actin cytoskeleton [43]. It is possible that some of the effects observed in immature astrocytes in response to BMPs could be mediated through a non-canonical pathway. More work is needed to fully elucidate and confirm the relevant intracellular signaling cascades.

Dissecting BMP signaling in astrocyte maturation *in* *vivo* is further complicated by the fact that there are multiple sources of BMPs in the developing brain [44]. In addition, our *in* *vitro* data demonstrates that immature astrocytes are capable of responding to many BMP family members. During early postnatal development, when astrocytes are maturing, various cell types express relatively high levels of at least one BMP that was capable of modulating immature astrocyte biology *in* *vitro*. This includes oligodendrocyte precursor cells, newly formed oligodendrocyte, neurons, and pericytes [14]. It will be interesting for future work to parse out which BMPs and sources are most important for astrocyte maturation in the intact brain. This includes confirming the survival effect of BMP signaling on immature astrocytes *in* *vivo*, which was not directly examined in this study.

### BMP is a signal that dissociates astrocyte survival from proliferation

*In vivo*, astrocyte proliferation and apoptosis are essentially complete by P14, and mature astrocytes exhibit limited division in healthy brains [3], [45]. How astrocyte number is controlled has remained a mystery. One possible mechanism is the existence of a signal that restricts astrocyte proliferation at the correct time and location. BMP signaling in immature astrocyte has a strong trophic effect, yet it simultaneously halts proliferation, which makes BMP an intriguing signal.

However, *in* *vivo*, smad1/5/8 pathway activation did not alter cortical astrocyte proliferation rates at P3 based on ki67 staining. This finding suggests that perhaps other cues are present *in* *vivo* that can modify astrocyte interpretation of BMP signaling or dominantly influence proliferation. Environmental modulation could perhaps lead to a delay between observed smad1/5/8 pathway activation and the cessation of astrocyte division.

Although developmental astrocyte proliferation is actively attenuated, astrocytes in the adult CNS are not post mitotic. In response to certain disease states or injuries, mature astrocytes can resume proliferation [24], [46]. Importantly, even after prolonged exposure to inhibitory BMP signal *in* *vitro*, astrocytes readily resume dividing when put in a permissive environment. Further studies of BMP-induced inhibition of mitosis could yield insight into the intracellular mechanisms underlying reversible control of astrocyte proliferation.

### Astrocyte heterogeneity

Research has revealed that astrocytes are a remarkably heterogeneous group of cells that vary in multiple descriptive and functional properties [2], [47], [48]. For example, the resting expression level of the classic astrocyte marker GFAP is vastly different across regions of the CNS [23]. In the adult brain, GFAP is expressed highly by most fibrous astrocytes but is only seen in a small percentage of protoplasmic cortical astrocytes [49]. We found that during postnatal cortical development, layer 1 astrocytes displayed the highest and longest duration of smad1/5/8 pathway activation. These cells were also the cortical astrocytes that most strongly expressed GFAP. So, perhaps BMP signaling could play a role in the developmental origins of astrocyte GFAP heterogeneity. This hypothesis could be examined further in future experiments by comparing smad1/5/8 pathway activation in developing grey and white matter astrocytes.

### Regulation of EGFR by BMP signaling

The slowing of proliferation as astrocytes mature could also be achieved by modulation of their intrinsic response to, or reliance on, external cues. For example, although EGFR signaling is important for many aspects of early astrocyte development [33], [34], [50], [51], it is sharply downregulated after the first postnatal week [10],[12],[36]. We show here that EGFR protein is not expressed by P14 cortical astrocytes, which changes their receptivity to mitogenic EGFR ligands and could contribute to the attenuation of astrocyte proliferation. Despite the fact that EGFR downregulation is a well-characterized step in astrocyte maturation, how it is signaled and coordinated remains unclear. We found that BMP signaling in immature astrocytes *in* *vitro* is sufficient to directly and robustly downregulate EGFR expression, even in the presence of other mitogens. This is the first report of an extrinsic factor that can directly modulate astrocyte EGFR expression. Our findings do not support, however, the conclusion that BMP signaling via smad1/5/8 is sufficient for developmental downregulation of astrocyte EGFR *in* *vivo,* and raises the question of what other signaling pathways could be involved.

Independent of astrocyte maturation, the EGFR result deepens our general knowledge of receptor transmodulation, which is when the expression of one cell-surface cytokine receptor is modulated by another cytokine. This cross-communication between different signaling systems is important for integrating diverse stimuli and creating effective feedback mechanisms. A study in NPCs reported that BMP signaling could antagonize the EGFR pathway [51], but complete receptor downregulation directly in response to BMP signaling is novel. It would be interesting to uncover the mechanism mediating cross talk between the BMP and EGFR pathways in astrocytes and to see if this transmodulation is applicable in other cell types.

### BMP signaling in injury and disease: gliosis and glioma

In the diseased or injured brain, astrocytes can respond by undergoing gliosis, and in severe cases, forming a glial scar [46]. Reactive astrocytes are characterized by changes in their molecular expression, including GFAP upregulation, and morphology. Recently, it has become clear that proliferation of reactive astrocytes is rarely a substantial component of glia scar formation [24], [52]. BMP signaling and smad1/5/8 pathway activation have been previously implicated in gliosis [53]. In the spinal cord, cell-autonomous roles for Bmpr1a and Bmpr1b in mediating reactive astrocyte hypertrophy have been identified [54]. We found that BMP stimulation of immature astrocytes *in* *vitro* upregulates GFAP expression without increasing proliferation, which aligns with a basic reactive astrocyte phenotype. Also, preliminary gene chip data (unpublished data) suggests that some additional reactive genes, including lipocalin-2, are upregulated by BMP5. The exact impact of BMP signaling on gliosis remains unclear, but multiple lines of evidence suggest a possible role.

The most common brain tumor in humans is astrocyte-derived glioblastoma multiforme (GBM), which is typically highly proliferative and very malignant. Amplification of the EGFR gene is seen in 40 to 70% of primary GBMs [55]. Gene amplification and some additional type of mutations all cause overexpression of EGFR protein in the tumor cells. Consequently, EGFR has been a prime target for therapeutic intervention [56]. We have shown that BMP signaling can effectively halt astrocyte proliferation, at least partially by downregulating EGFR expression. Similarly, BMP4 has been found to dramatically reduce both proliferation of human GBM cells *in* *vitro* and their tumorigenicity *in* *vivo* [57]. Our data in primary astrocytes suggests a possible mechanism mediating the effect of BMP4 on GBMs: direct downregulation of EGFR. Elucidation of the mechanism underlying the crosstalk between BMP signaling and EGFR in astrocytes could be applicable to GBM, and potentially aid in the development of therapeutics.

## Experimental Procedures

### Ethics Statement

All procedures were approved by the Stanford University Administrative Panel on Laboratory Animal Care (APLAC) under protocol #10726. Rats were euthanized with CO2 or rapid decapitation (neonates). Mice were anesthetized with ketamine and xylazine.

### Purification and culture of rat astrocytes

Postnatal rat astrocytes were purified by immunopanning from six to ten Sprague-Dawley (Charles River) rat forebrains and cultured as previously described [10]. Briefly, cortices were enzymatically then mechanically dissociated to generate a single cell suspension that was incubated on successive negative immunopanning plates to remove microglia, endothelial cells, and OPCs before positively selecting for astrocytes with an ITGB5-coated panning plate. Isolated astrocytes were cultured in a defined, serum-free base media containing 50% neurobasal, 50% DMEM, 100 units of penicillin, 100 µg/ml streptomycin, 1 mM sodium pyruvate, 292 µg/ml L-glutamine, 1X SATO and 5 µg/ml of N-acetyl cysteine as previously described [10].

### Survival Assays

Acutely isolated astrocytes were plated at 2,000 cells/well in poly-d-lysine-coated (PDL) 24-well plates in base media with 0.5 µg/ml aphidicolin (Sigma A0781). Unless otherwise noted, candidate cytokines were individually added at the following concentrations: 5 ng/ml HbEGF (Peprotech); 100 ng/ml IGF2 (R&D Systems); 100 ng/ml CXCL12 (R&D Systems); 50 ng/ml BDNF (Peprotech); 10 ng/ml NT3 (Peprotech); 10 ng/ml FGF1, FGF2, FGF8, and FGF9 (Peprotech); 100 ng/ml BMP2, BMP4, BMP5, BMP6, BMP7, BMP10, BMP15, GDF3, and GDF5 (R&D Systems); 250 ng/ml Activin A (R&D Systems); 100 ng/ml Nodal (R&D Systems); 100 ng/ml TGFB2 (R&D Systems). Survival was assayed at 3DIV using a Live/Dead kit in which calcein AM stains live cells green and ethidium homodimer stains dead cells red (Life Technologies L3224). At least 3 independent experiments were conducted for each condition. For each experiment, 3 non-overlapping 20x fields per well were quantified in triplicate wells. Significance determined using one-way ANOVA with Dunnett correction.

### RT-PCR

Total RNA was isolated from P7 rat brain or acutely purified astrocytes using the RNeasy Mini Kit (Qiagen). 500 ng of total RNA was converted to cDNA using SuperScriptIII (Invitrogen) and equivalent volumes of the RT reaction were PCR amplified using Platinum Taq (Invitrogen).

### Western Blotting

Protein samples were collected at 4C in RIPA buffer containing Complete Protease Inhibitor Cocktail (Roche) and Halt Phosphatase Inhibitor Cocktail (Thermo Scientific). Sample concentrations were determined with the BCA assay (ThermoScientific) and equivalent amounts of total protein were loaded onto 4–15% Tris-HCl gels (Bio-Rad). HT-1080 Control Cell Extracts (Cell Signaling 12052) were used as positive controls for smad2/3 activation and actin levels were used as loading controls. Following electrophoresis, proteins were transferred to Immobilon-P (EMD Millipore) membranes. Blots were probed overnight at 4C with 1∶1000 rabbit-anti-Smad5 (Cell Signaling 12534), 1∶1000 rabbit-anti-Smad2 (Cell Signaling 5339), 1∶1000 rabbit-anti-Phospho-Smad1/5/8 (Cell Signaling 9511), 1∶1000 rabbit-anti-Phospho-Smad2 (Cell Signaling 3108), 1∶1000 rabbit-anti-EGFR (Cell Signaling 2232), and 1∶1000 rabbit-anti-β-actin (abcam 8227). Blots were then incubated with HRP-conjugated secondary antibodies at 1∶10,000 for 1 hour at room temperature and developed using ECL Prime Western Blotting Detection Reagent (GE Healthcare). Visualization and imaging of blots was performed with a FluorochemQ System (ProteinSimple).

### *In Vitro* Immunostaining and Morphology Analysis

Cultured astrocytes were fixed with 4% paraformaldehyde (PFA) for 10 min at 3DIV. Cells were blocked in 50% goat serum and immunostained with 1∶1000 rabbit-anti-GFAP (Dako Z0334) for 2 hours at room temperature followed by an Alexa594 conjugated secondary antibody. Other antibodies were used at the following concentrations: rabbit-anti-AQP4 (Sigma A5971) at 1∶1000, mouse-anti-S100β (Sigma S2532) at 1∶200, rabbit-anti-Sox2 (abcam 97959) at 1∶1000, mouse-anti-Nestin (BD Biosciences 556309) at 1∶1000, mouse-anti-HuC/HuD (Life Technologies A21271) at 1∶10, mouse-anti-Tuj1 (Sigma T8660) at 1∶1000, rabbit-anti-NG2 (Millipore AB5320B) at 1∶500 and rat-anti-MBP (abcam 7349) at 1∶300. For morphology analyses, cell membranes were then labeled by incubation with 1x HCS CellMask Green (Life Technologies H32714) in PBS for 30 min. Cells were mounted in DAPI Fluoromount-G (Southern Biotech) and duplicate wells from 3 independent experiments imaged at 10x on an inverted Zeiss microscope. Morphology and process length were determined from the CellMask membrane staining images and quantification done using the Sholl Analysis plugin in ImageJ [22]. Primary process number was calculated at a radius of 20 µm from the center of the cell body. Significance determined using one-way ANOVA with Tukey correction.

### Clonal Analysis

Astrocytes were plated at 5,000 cells/dish in PDL coated 10 cm culture dish in minimal media. Clone size was counted using a 10x objective on a phase microscope every 24 hours for 7DIV. For reversibility experiments, astrocytes were cultured in BMP5 containing media for 7DIV, then lifted by trypsinization and replated at 5,000 cells per 10 cm dish. Clone size was counted for 7 days starting at 8DIV. Significance determined using one-way ANOVA with Tukey correction.

### Immunohistochemisty

Aldh1l1-eGFP were perfused with PBS followed by 4% PFA. Brains were postfixed in 4% PFA overnight at 4C, equilibrated in 30% sucrose, mounted in O.C.T. (Tissue-Tek) and sectioned into 10 µm thick sagittal cryosections. Sections were blocked with 10% goat serum in 0.1% PBST then stained with 1∶1000 chicken-anti-GFP (EMD Millipore AB16901), 1∶1500 rabbit-anti-Psmad1/5 (Cell Signaling 9516), 1∶300 rabbit-anti-ki67 (Cell Signaling 12075S) and 1∶2000 mouse-anti-GFAP (Sigma G3893) antibodies overnight at 4C. Alexa594, 568, 647 or 488 conjugated secondary antibodies were then added at 1∶1000 for 2 hrs at room temperature and sections were mounted in DAPI Fluoromount-G (Southern Biotech). Image acquisition was done at 20x on a Zeiss Axiocam microscope and expression quantified using ImageJ. Simultaneous staining with rabbit-anti-p-smad1/5 and rabbit-anti-ki67 was done using a sequential technique. P-smad1/5 staining and visualization with an Alexa568 conjugated secondary was completed first. Then, the sections washed repeatedly and stained with Alexa647 conjugated ki67. Co-localization of eGFP+ astrocytes with ki67 and p-smad1/5 at P3 was examined in 3 independent brains. P-smad1/5 colocalization with eGFP+ astrocytes was examined in two independent mouse brains for each postnatal time point. Significance was determined using a students t-test.

## Supporting Information

Figure S1 **Multiple BMPs activate the smad1/5/8 pathway, promote a process-bearing morphology and inhibit proliferation in purified astrocytes.** A. Western blots of purified astrocytes cultured in HbEGF then treated with 50 ng/ml of BMP4 or BMP6 for 2 hours show robust activation of the smad1/5/8 pathway (p-smad1/5/8). Actin bands confirm equal protein loading. B–D. Images of purified astrocyte morphology using Calcien AM staining. Astrocytes were cultured for 3DIV in HbEGF+BMP4 (B), HbEGF+BMP6 (C), or HbEGF+BMP5 (D). Scale bars are 50 µm. E. The proliferative capabilities of purified astrocytes in BMP4, BMP6, and HbEGF+BMP6 were quantified *in* *vitro* over 7 days using clonal analysis. Average clone size was calculated for all media conditions. Error bars represent SEM.
(TIF)

Figure S2 **Immunostaining of astrocyte cultures at 3DIV for markers of other cell types.** A**–**L. Purified astrocytes were cultured for 3DIV in HbEGF or BMP5 and the expression of markers for neural progenitors (Sox2 and Nestin), neurons (HuC/D and Tuj1), and oligodendrocyte-lineage cells (NG2 and MBP) were examined by immunostaining. All cultures were also stained with GFAP. Scale bars are 50 µm.
(TIF)
