# Supplementary figures and images for: BMP Signaling in Astrocytes Downregulates EGFR to Modulate Survival and Maturation
Source: PLoS One. 2014 Oct 17;9(10):e110668. doi: 10.1371/journal.pone.0110668 (PMC4201562; doi:10.1371/journal.pone.0110668)

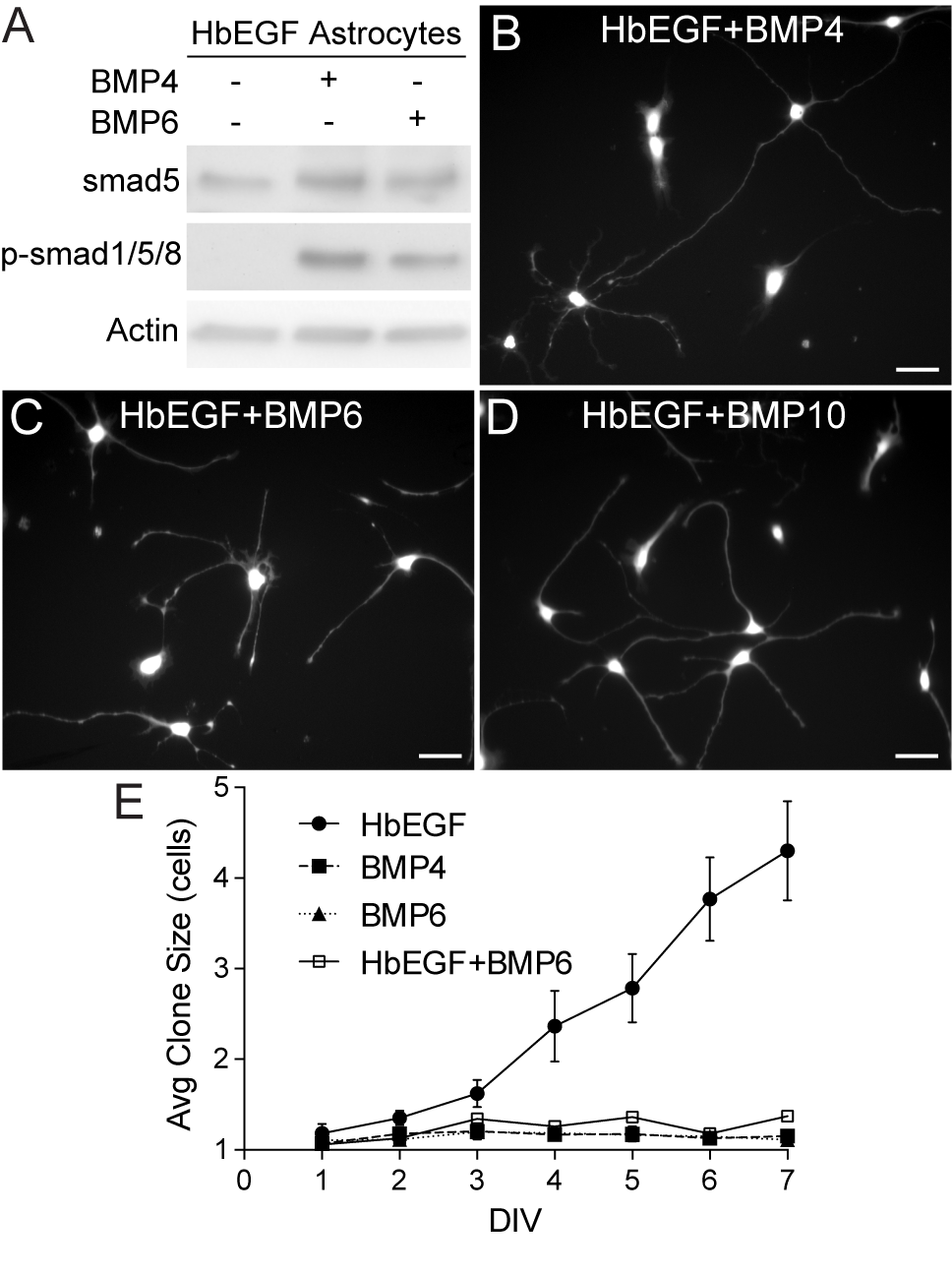

Supplement: Figure S1 — Multiple BMPs activate the smad1/5/8 pathway, promote a process-bearing morphology and inhibit proliferation in purified astrocytes. A. Western blots of purified astrocytes cultured in HbEGF then treated with 50 ng/ml of BMP4 or BMP6 for 2 hours show robust activation of the smad1/5/8 pathway (p-smad1/5/8). Actin bands confirm equal protein loading. B–D. Images of purified astrocyte morphology using Calcien AM staining. Astrocytes were cultured for 3DIV in HbEGF+BMP4 (B), HbEGF+BMP6 (C), or HbEGF+BMP5 (D). Scale bars are 50 µm. E. The proliferative capabilities of purified astrocytes in BMP4, BMP6, and HbEGF+BMP6 were quantified in vitro over 7 days using clonal analysis. Average clone size was calculated for all media conditions. Error bars represent SEM. (TIF) [file pone.0110668.s001.tif]

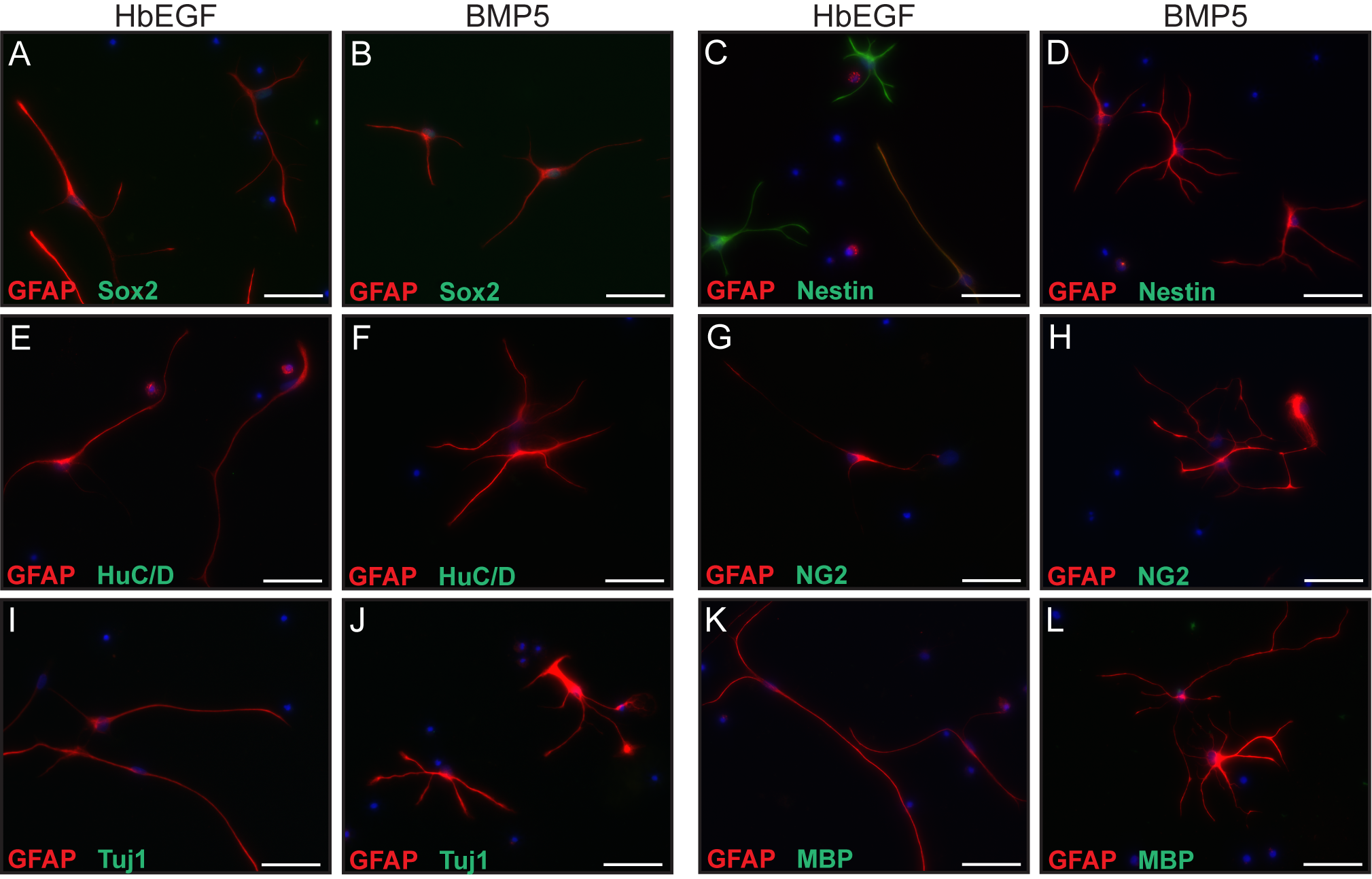

Supplement: Figure S2 — Immunostaining of astrocyte cultures at 3DIV for markers of other cell types. A–L. Purified astrocytes were cultured for 3DIV in HbEGF or BMP5 and the expression of markers for neural progenitors (Sox2 and Nestin), neurons (HuC/D and Tuj1), and oligodendrocyte-lineage cells (NG2 and MBP) were examined by immunostaining. All cultures were also stained with GFAP. Scale bars are 50 µm. (TIF) [file pone.0110668.s002.tif]
